# A novel systems solution for accurate colorimetric measurement through smartphone-based augmented reality

**DOI:** 10.1371/journal.pone.0287099

**Published:** 2023-06-15

**Authors:** Guixiang Zhang, Shuang Song, Jenny Panescu, Nicholas Shapiro, Karen C. Dannemiller, Rongjun Qin

**Affiliations:** 1 Department of Civil, Environmental and Geodetic Engineering, The Ohio State University, Columbus, Ohio, United States of America; 2 Department of Electrical and Computer Engineering, The Ohio State University, Columbus, Ohio, United States of America; 3 Geospatial Data Analytics Lab, The Ohio State University, Columbus, Ohio, United States of America; 4 Institute for Society and Genetics, University of California, Los Angeles, Los Angeles, California, United States of America; 5 Environmental Health Sciences, The Ohio State University, Columbus, Ohio, United States of America; 6 Sustainability Institute, The Ohio State University, Columbus, Ohio, United States of America; 7 Translational Data Analytics Institute, The Ohio State University, Columbus, Ohio, United States of America; Sichuan University, CHINA

## Abstract

Quantifying the colors of objects is useful in a wide range of applications, including medical diagnosis, agricultural monitoring, and food safety. Accurate colorimetric measurement of objects is a laborious process normally performed through a color matching test in the laboratory. A promising alternative is to use digital images for colorimetric measurement, due to their portability and ease of use. However, image-based measurements suffer from errors caused by the non-linear image formation process and unpredictable environmental lighting. Solutions to this problem often perform relative color correction among multiple images through discrete color reference boards, which may yield biased results due to the lack of continuous observation. In this paper, we propose a smartphone-based solution, that couples a designated color reference board with a novel color correction algorithm, to achieve accurate and absolute color measurements. Our color reference board contains multiple color stripes with continuous color sampling at the sides. A novel correction algorithm is proposed to utilize a first-order spatial varying regression model to perform the color correction, which leverages both the absolute color magnitude and scale to maximize the correction accuracy. The proposed algorithm is implemented as a “human-in-the-loop” smartphone application, where users are guided by an augmented reality scheme with a marker tracking module to take images at an angle that minimizes the impact of non-Lambertian reflectance. Our experimental results show that our colorimetric measurement is device independent and can reduce up to 90% color variance for images collected under different lighting conditions. In the application of reading pH values from test papers, we show that our system performs 200% better than human reading. The designed color reference board, the correction algorithm, and our augmented reality guiding approach form an integrated system as a novel solution to measure color with increased accuracy. This technique has the flexibility to improve color reading performance in systems beyond existing applications, evidenced by both qualitative and quantitative experiments on example applications such as pH-test reading.

## Introduction

The ability to quantify the colors of objects has had many applications in recent years, including calibrating digital screens [1], counting cells [2], pH detection [3], the inspection of contaminated water [4], at-home food colorant measurement [5], colorimetric enzymatic assay [6], and analysis of sweat [7, 8] and skin [9, 10]. For example, it can be used to measure the total loss of sweat, the rate of sweating, the temperature of sweat, and the concentrations of electrolytes and metabolites in sweat such as chloride, glucose, and lactate [7]. Additionally, accurate color measurement can be used for analyzing the skin lesions such as melanoma and erythema in canine skin [9].

**Fig 1:**
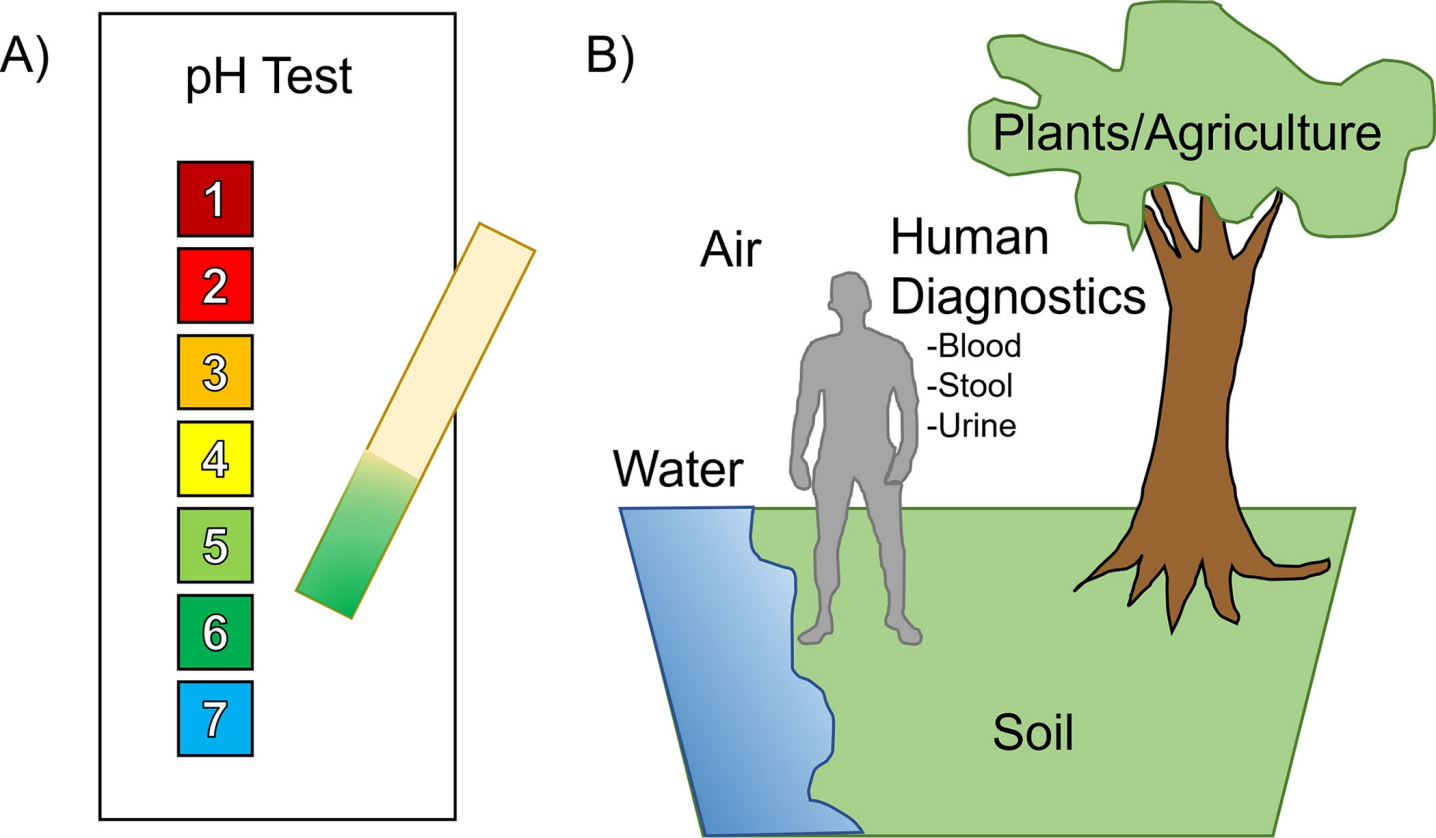
Examples of color-changing test kits. (A) A pH test kit: the pH test paper turns blue when exposed to a weak acid solution. (B) Other examples of properties that can use a color-changing test kit to detect.

Color-changing test kits can be developed to detect properties of interest in a wide range of media, such as water, air, blood, urine, and others [11] (Fig 1). Usually, these tests require a human to read the colors in comparison to a reference color chart to determine values and positivity (Fig 1A). The results of human reading may introduce large margins of error due to the biological differences in individuals’ color perceptions [12–14]. Up to now, the most rigorous way of identifying the colors of an object is to undergo a laborious color matching test [15] where an operator manually adjusts a mixture of RGB (red, green, and blue) reference light, to perceptually match the color of an object. Reading colors using cameras can be a much less labor-intensive process, and the underlying rationale is to interpret the reflectance of surface material (or the ambient color of the object) from the colors. Due to the varying environment lighting and shadowing effect at data collection, colorimetric measurement requires a process to calibrate the cameras for color reading. This calibration is often performed in a controlled environment (e.g., in the laboratory) [16] where the intensity and the direction of the lighting are known (such as in light chambers [17–19]).

Nowadays smartphone is regarded as the most commonly used sensor suites [20]. It integrated not only high-resolution cameras but also strong processing powers to facilitate any needed onboard data processing, which makes it a promising platform for accurately quantifying colors at a low cost. As mentioned earlier, reading colors from the camera is a non-trivial task, as it requires a calibration process to overcome the difference of the image sensors and environmental lighting that are otherwise only possibly done in a controlled environment. Typical solutions use a non-parametric approach and require either a one-time or per-capture calibration.

For example, Kong *et al*. [2] performed a per-capture calibration for single-color shifts using continuous smartphone LED as the dominant light source and rescaling the color with the white and black background. Although the results showed that this method can compensate for ambient lighting conditions and reduce variances among different devices, it is limited when the LED is not the dominant light source. In addition, the multiple steps of the process need many manual operations such as aligning the lights, picking, and reading background color for calculation may produce errors and variations in the final results. To reduce calibration effort, Nixon *et al*. [21] proposed to use a combination of per-sensor calibration and a simpler version of per-capture calibration to obtain device-independent colorimetric readings. The per-capture calibration requires a perfectly aligned image pair with and without a flashlight, to subtract environmental lighting; the per-sensor calibration adopts a collocated discrete color board (with known color values) to correct the sensor-specific tones to achieve “device independence”. Solmaz *et al*. [22] proposed to use a data-driven approach, *i*.*e*., learning from examples using a machine learning model to classify colorimetric tests. This approach cannot predict continuous values and, like many learning-based methods, may suffer from generalization problems [23]. Therefore, optimal solutions must work under general lighting conditions with minimal capture efforts.

In this paper, we propose a novel smartphone-based solution to perform accurate colorimetric measurements of environmental objects. The proposed solution follows a per-capture calibration scheme while proposing a completely new color reference board to capture the heterogeneity of the environmental light to allow spatial varying color corrections. Moreover, the existing methods assume the materials are non-reflective (*i*.*e*., assuming the Lambertian Model [24]), our solution implements a scheme to address this caveat to alleviate reflective effects: based on the smartphone camera, our solution implements an augmented reality-based approach, *i*.*e*., a tracking algorithm visualizing moving directions on the camera feed, to guide the users taking the images at a consistent angle to reduce the non-Lambertian effects. In general, the proposed solution is highly integrative, consisting of 1) a color reference board, 2) an augmented reality-based image capture module, 3) and a color correction algorithm. Our system bears a high potential for use in field sampling, telemedicine, and citizen science in addition to lab settings and dramatically increases resolution beyond current methods that rely on human observation. We demonstrate the utility of our solution by improving the performance of reading pH test stripes compared to reading by the human eye.

The rest of the paper is organized as follows. In the Section “Materials and Methods”, the details of the approach are described including the design of our color reference board, our augmented reality-based image acquisition module, and our color correction algorithm. Two simulation experiments and two physical experiments are described in the Section “Experiments” to demonstrate the capabilities of our approach. Methods and experiments are summarized in the Section “Conclusions”.

## Materials and methods

In this section, we describe the design concept of our color reference board and our data processing algorithms. We implement our algorithms in an iOS smartphone application to better demonstrate the power of augmented reality-based image capturing.

**Fig 2:**
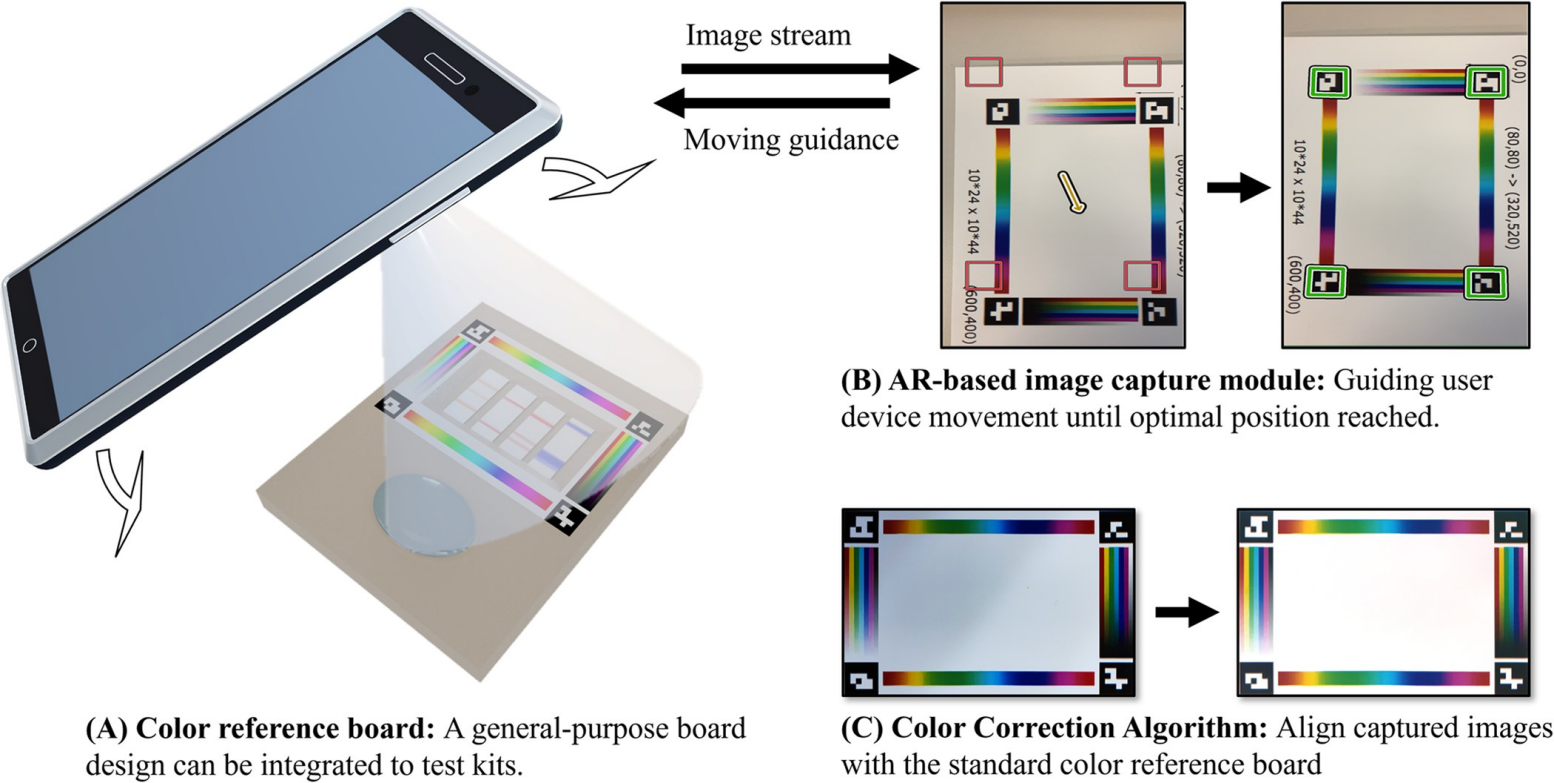
The main modules of our system. (A) a general-purpose color reference board that can combine with other test kits or derive new kits; (B) our proposed augmented reality-based image capture module that continuously provides movement guidance to users leading to an optimal position for best capturing quality; (C) our proposed color correction algorithm that aligns images taken under natural illumination to the standard color reference board which leads to other post-processes (*e*.*g*., automatic reading).

Our proposed colorimetric measurement system includes a machine-friendly color reference board and a smartphone application (Fig 2). It consists of three modules: the first module refers to a machine-friendly color reference board, which includes reference colors for correction, and markers for marker localization. The board is flexible and can be adapted to various existing test kits or be integrated into new test kits (see Section “Design of the color reference board”); the second module refers to our augmented reality-based image capture system, which efficiently processes the camera video feeds to automatically localize color reference board and compute the position and orientation of the smartphone. This information is used to guide the users in real-time to place the camera at the optimal position to take images at the best angle (see Section “Augmented reality-based image capture guidance module”); the third module refers to our color correction algorithm, which corrects the color of the objects using the color from the standard color reference board (see Section “The color correction algorithm”). In the following, we will describe each of these modules in detail.

### Design of the color reference board

**Fig 3:**
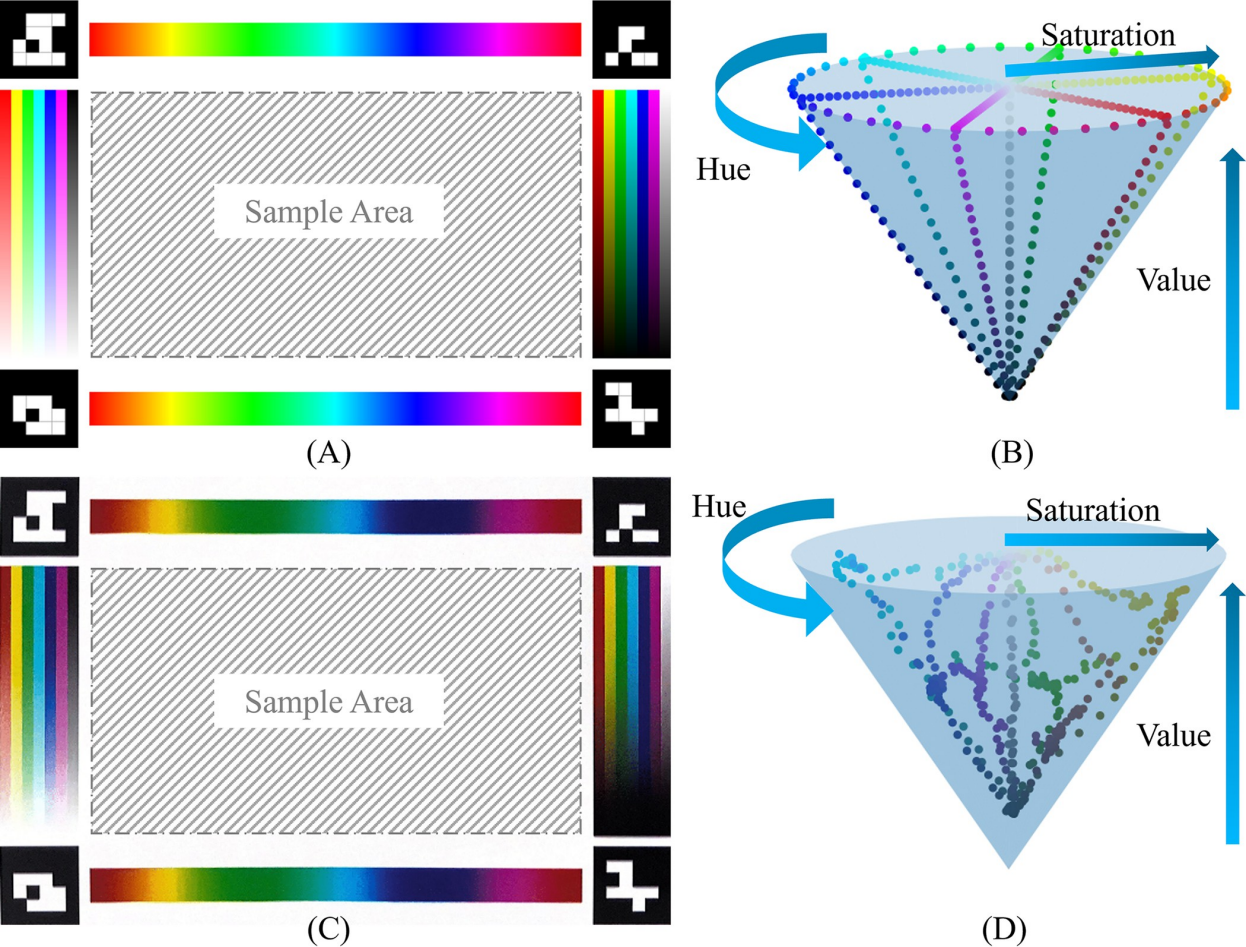
The standard color reference board and the visualization of the sample point in HSV color space. (A) The source file of the color reference board. (B) Source color samples in HSV space. (C) The digitalized file of the color reference board. (D) Digitalized color samples in HSV space.

The design of our color reference board is critical to ensure accurate color determination (Fig 3A). In this board, we employ the ArUco [25, 26] markers at each corner of the border, since the ArUco markers are proven robust for image-based detection, and have been widely applied in the field of computer vision and robotics. Additionally, ArUco possesses the following advantages in our color reading applications: first, the white/black pattern is robust to various viewing angles and non-uniform illumination; second, based on its binary coding, each ArUco marker can be uniquely identified to represent a different corner of the reference board to facilitate estimating orientations; third, ArUco code advances its alternative—QR code [27] by providing redundant information in its coding, such that the marker is detected when only partial information is present; fourth, ArUco is open-source and its implementation can be easily found and used through well-known computer vision libraries (i.e., OpenCV [28]). Between these markers, we placed the reference color stripes along each side, and the central region of the board is used to host the samples. To build an accurate color correction algorithm, the color on the stripes was designed to cover as many visible spectrums as possible (ca. 380-700nm in wavelength). In the study, the color stripes on the sides of the reference board are rendered by regularly sampling the full (linear) color space determined by this Hue-Saturation-Value (HSV) color model [29] (Fig 3B): the stripes at the top and bottom of the board are generated by regularly sampling at an interval of *H*∈[0,1], while keeping the other two components as constants *S* =1, *V* = 1. The left and right stripes are generated in a similar way with (*H*∈[0,1], *S*∈[0,1], *V* = 1) and (*H*∈[0,1], *S* = 1, *V*∈[0,1]) respectively. All the stripes described above serve as the control colors (stripes with known color values in HSV space). All other colors in the space are supposed to be the stable linear combination/interpolation of those sample points surrounding the entire color space. In addition, compared to the additive RGB color model, the HSV color model interprets the color close to human perception (with perceptual attributes, hue, saturation, and value). Although in some other papers, researchers used CIELab color space [30] which is more perpetually linear and covers the entire gamut (range) of human color perception, only part of the space is useful for processing the image in the computer. The 3D geometry of those usable colors is not regular and difficult to use the limited number of color stripes on our test board to represent. In contrast, the HSV color model has a regular cone geometry, so we can select color stripes and sample reference points on them that can not only cover the entire useful color space using limited space on the color reference board but also simulate how human reads color. Using the captured colors (distorted from the standard colors), the color correction algorithm can model the color distortion mathematically to correct the image content. In the Section “Impact of different color stripe patterns and color correction models on the reference board”, we proposed different designs of the color reference board by changing the pattern of color stripes and compared them by analyzing color correction accuracy. We found that the design with full HSV color space coverage outperforms other patterns which partially covered HSV color space.

In practice, a standard color reference board with the theoretically correct color can be difficult to achieve, as the varying constraints of printers and their inks may easily distort the colors appearing on reference boards. The color displayed in the source files and the printed color are notably different as can be seen in Fig 3A and 3C. If we still correct the colors referring to the theoretically designed colors, the model will need to involve complicated modeling of the color distortion process of the certain printer, making the problem intractable. In our experiment, instead of taking the theoretical color values appearing in the standard color reference board, as shown in Fig 3A and 3B, we digitalized the color reference board from the printed material by scanning with a common smartphone app and reassigning the color values of the standard color reference board based on the digitalized (scanned) printed color reference board as the intermediate color system. Empirically we found this to be effective to adapt the standard color reference board by users with different printers.

### Augmented reality-based image capture guidance module

**Fig 4:**
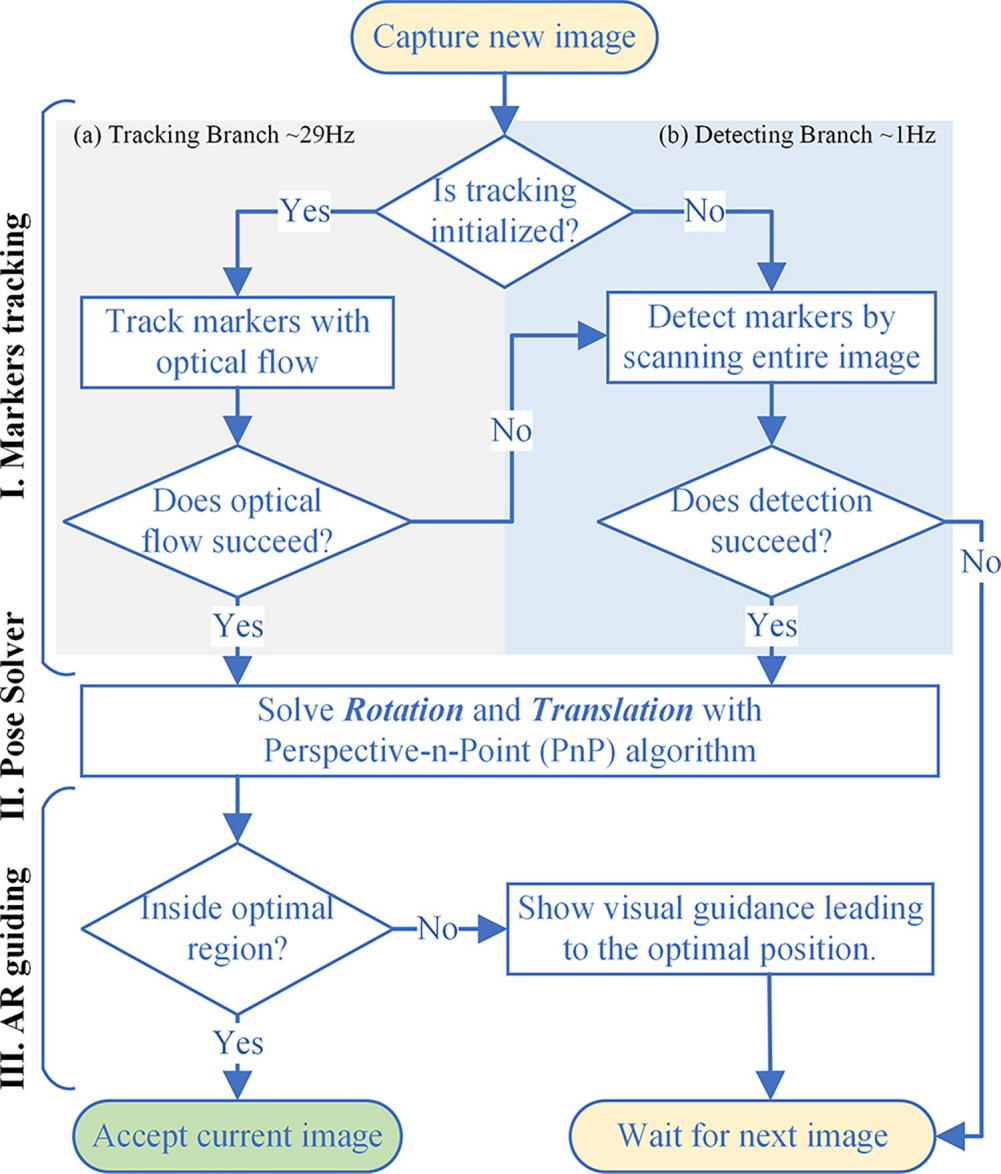
The workflow of our augmented reality-based image capture module.

We design a “human-in-the-loop” process to standardize the image capture practice to alleviate possible errors due to inconsistent collection angles and illumination. An augmented reality-based module is proposed and implemented to guide the users to acquire images that are consistent in viewing angle. With the ArUco markers, we can define an optimal photo-taking pose in 3D space related to our color reference board and guide users to approximate the same collection angle. By fixing the capturing angle as much as possible, the system inherently separates possible non-Lambertian surfaces (such as reflective surfaces). The algorithm starts by estimating the position of the camera (location and facing/orientation) when the users attempt to capture the image. This is done by computing the difference between the estimated orientation and the desired one. A correcting direction appearing will be computed and visualized as an arrow in the center of the image frame (example shown in Fig 2B). This arrow guides the user to adjust the orientation of the camera until the arrow is minimized, followed by an automatic shuttering to take the desired image. A more detailed algorithmic flow is shown in Fig 4, which consists of three components. 1) Marker tracking submodule, which keeps the markers in track as the user moves and it can provide up to 16 very stable key points for localization, noting that only minimally three points are needed. 2) Our pose solver, which takes the 16 key points from the tracking module, to compute the relative position and orientation of the camera using the 3D computer vision method, as mentioned in 1), the 16 points provide additional redundancy over the three minimally needed points to ensure robust and accurate pose estimation. 3) Our augmented reality-based guiding submodule, which serves as the final gatekeeper, decides whether or not to accept an image as the final candidate using the desired angle and position as the key criterion. In the following, we further elaborate on each submodule in the order of the processing sequence.

#### Markers tracking submodule

The goal of this submodule is to detect the pixel locations of up to 16 key points (4 corners of 4 ArUco markers) on a given image. The detection procedure we used in our system directly inherits existing implementations in open-source computer vision packages (e.g., OpenCV [28]). The parameters of these elementary processing algorithms have been carefully tweaked to twin the detection with the standard ArUco code. This detection procedure of ArUco markers is composed of a series of mature image processing methods. More details can be found in [31].

The process encounters limitations intrinsic to the mobile platform, such as its limited computing power and incurred battery use. Thus, executing ArUco detection for every frame can be suboptimal leading to not only a quickly drained battery but also degraded user experience due to the delay. To improve the time efficiency of our system, we proposed a lightweight tracking algorithm to speed up pixel localization by exploring the temporal coherence between video frames.

Since the video stream at image capture takes up to 30 frames per second (fps), it is expected that there will be minimal motion between temporally adjacent frames (with a time difference of only 33 milliseconds) at the scale of a few pixels. Therefore, in our algorithm, instead of detecting the key points for every frame, we first apply the key point detector to a single frame, followed by a local and fast pixel tracker called pyramid Lucas-Kanade optical flow [32] (PLK), to track these points. ArUco detection will be executed again on new frames if the PLK algorithm is failed to track all the key points due to out-of-boundary feature points or sudden camera motion. Additionally, the tracking algorithm might be subject to an accumulation of errors [33]. To ensure the robustness of the algorithm, the PLK algorithm will only be used when 16 key points are detected. With our hybrid detection and tracking method, we can improve the framerate for point detection and camera pose computation from 25 fps to 60 fps on our test phone, equivalent to a 140% improvement.

#### Pose solver submodule

**Fig 5:**
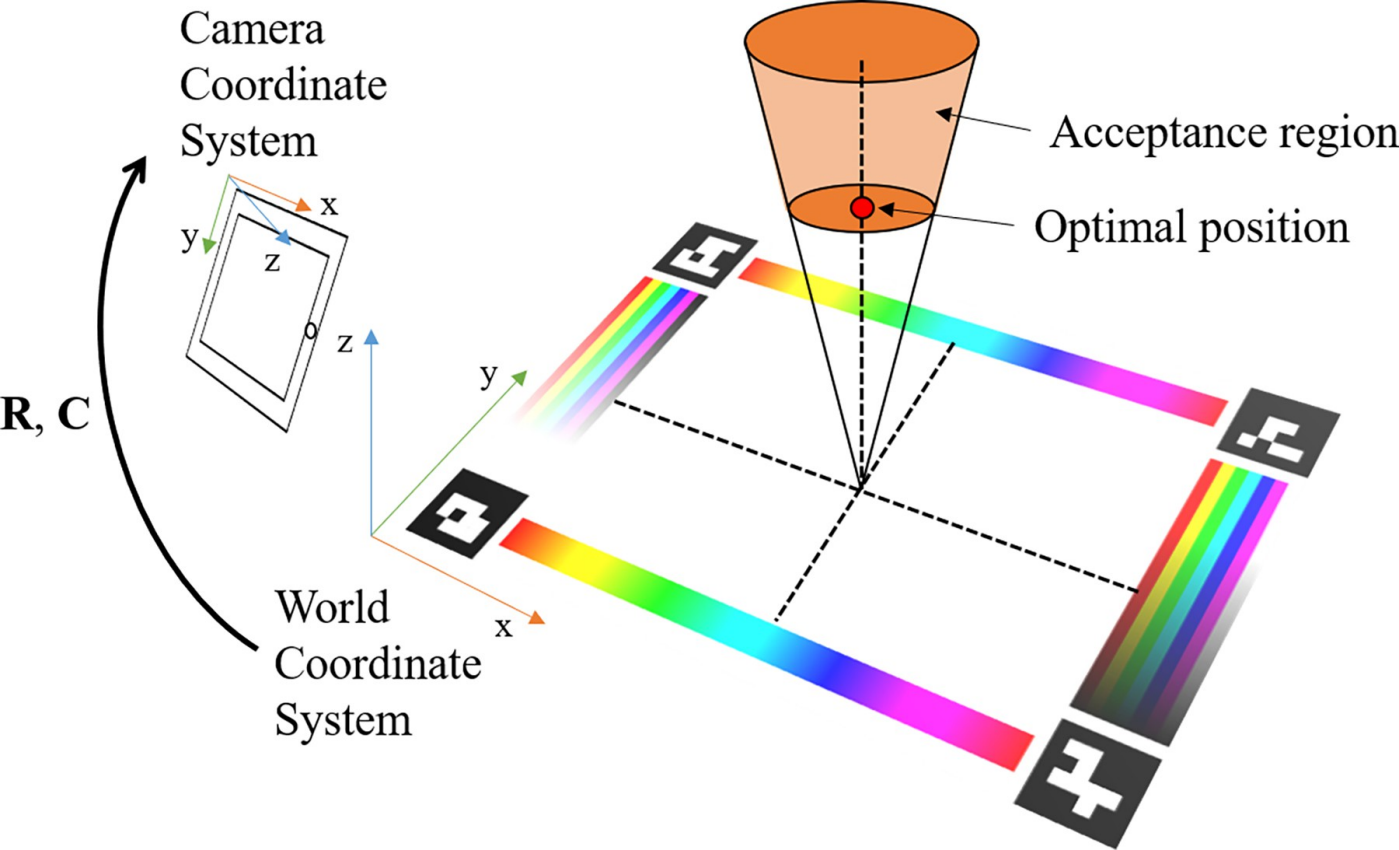
Definitions of coordinate frames in our system. The 2D image frame is composed of the x and y-axis of the camera coordinate system. The acceptance region refers to a region where camera placement is acceptable for image capture, determined as an area around the computed optimal camera placement position (discussed in Section “Augmented reality-based guiding submodule for image capturing”).

For each frame, we use the key points (either detected or tracked from the markers tracking submodule) as the input, to estimate the location of the image with respect to the world coordinate system (defined by the marker of the test badge, the printed color reference board in this paper). Given a key point location (denoted as *P*_*i*_∈ℝ^2^) from an image frame, the corresponding world location of the key point is also known to be on the test badge. Therefore, these key points can be used to recover the coordinate transformations between the image location and the predefined world location. As shown in Fig 5, the origin of the world coordinate system is defined at the bottom left corner of our color reference board, with axes x-right, y-up, and z-axis following the right-hand rule. The color reference board lies on the XOY plane of the world coordinate system, and the world coordinates of the key points are denoted as $P_{i}^{w}\in R^{3}$.

The relative pose (transformation) between the world coordinate system and image coordinate system can be interpreted as a homography [34] transformation (a delineating 3 × 3 projective transformation with eight degrees of freedom) including a rotation matrix (R) and a translation vector (C) as shown in Fig 5, and a pinhole camera intrinsic matrix (K). The transformation is described in Eq 1.

$$s[\begin{array}{c} P_{i}\\ 1 \end{array}]=K\times R\times (P_{i}^{w}-C)$$ (1)

where s denotes the scale factor in the similarity transformation, K∈ℝ^3×3^ denotes the pinhole camera intrinsic matrix, R∈ℝ^3×3^ denotes rotation transformation matrix and C∈ℝ^3^ denotes translation (camera center in world coordinate system). Readers may refer to [34] for details about the camera matrix. It is possible that a more complex model (i.e., non-linear transformation), may occasionally achieve better results if the camera lens is heavily distorted, but it would not be generalized to all cases, and will likely fit noises in the model.

In general, the intrinsic matrix K needs to be pre-calibrated [35] for every camera. Fortunately, most smartphone manufacturers provide calibration matrix and built-in lens distortion removal through their SDK (Standard Development Kit) [36, 37], which can be directly inherited used. Finally, we use the fast and robust Perspective-n-Point (PnP) algorithm [38] to solve rotation R and translation C from at least 3 $\left(P_{i},\;P_{i}^{w}\right)$ pairs.

#### Augmented reality-based guiding submodule for image capturing

We define the optimal camera position to be parallel to the color reference board, viewing from the top (shown as the red dot in Fig 5). With this orientation, the camera can capture the most details of the board and minimize perspective distortions. Some earlier works suggested 45 degrees as the optimal viewing angle [21], as it can minimize the specular reflectance and ambient light while using a flashlight as the light source sideways. However, our system using a viewing angle of 90 degrees favors Lambertian surfaces and maximizes the resolution that brings added benefits, see more details are introduced in the Section “Optimal viewing angle”.

The best capturing distance of the camera should be optimized based on the resolution and the coverage of the color reference board. Thus, using simple similarity triangles, we define the optimal height by Eq 2 as follows:

$$\frac{focal}{optimal\;height}=\min\left(\frac{W_{image}}{W_{board}},\frac{H_{image}}{H_{board}}\right)$$ (2)

where focal is the focal length in pixel unit. *W*_*image*_ and *H*_*image*_ are the width and height of the image plane in pixel unit. *W*_*board*_ and *H*_*board*_ are dimensions of the color reference board in the world unit. The optimal height is defined in the world unit.

We tolerate a small window around the optimal pose to allow a certain error margin for camera placement, which we call the acceptance region (shown in Fig 5), which we set as ϵ = 20 pixels around the optimal point. As for height tolerance, we allow the smartphone to be at 1.0 ~ 1.5 times optimal height, which yields images where the color reference board is properly located with sufficient resolution.

**Fig 6:**
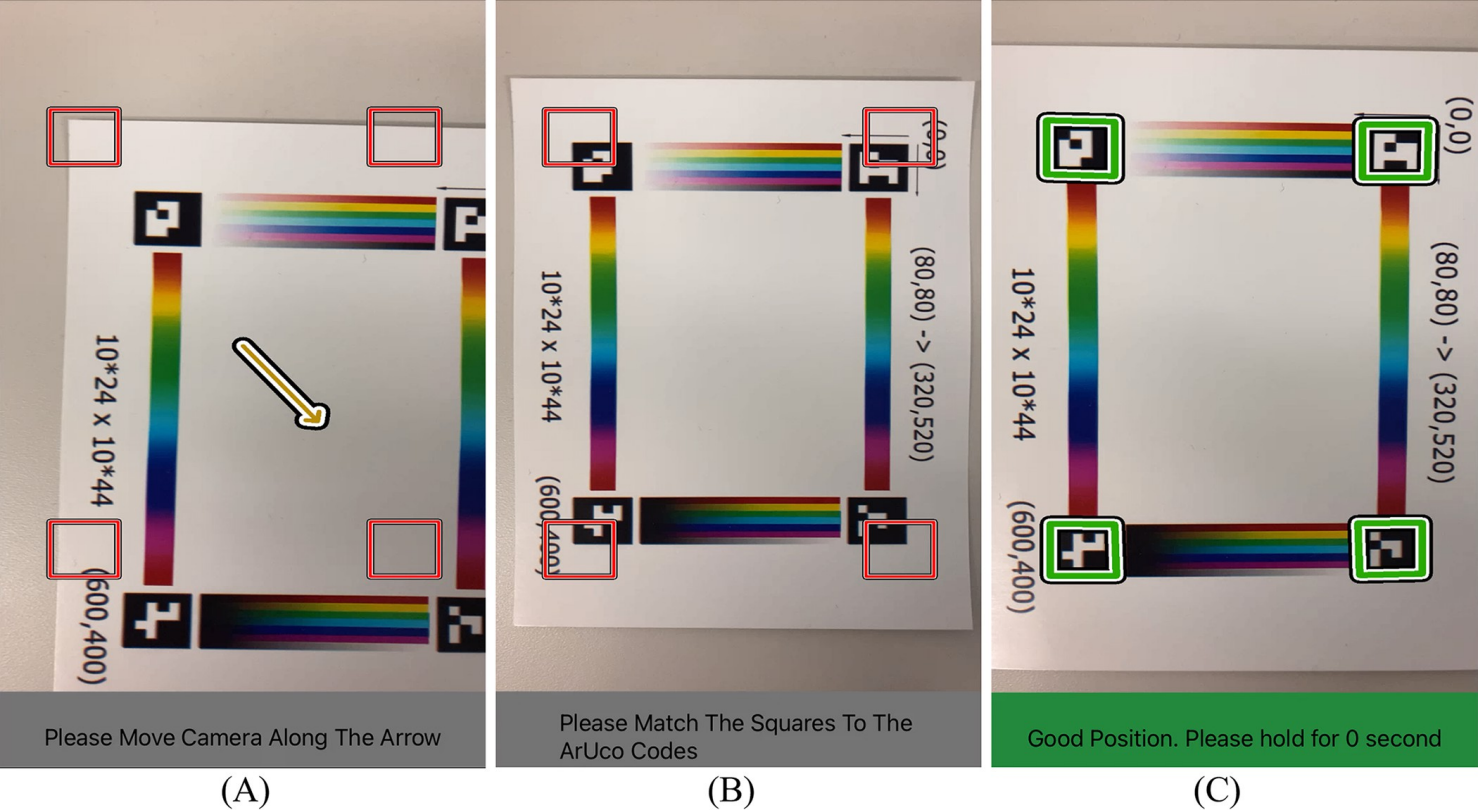
Augmented reality-based visual guidance module. (A) A screenshot of the App. at image capture, in which the users are asked to follow the yellow arrow to reach the optimal pose. (B) The frame is adjusted to align the square templates to the ArUco markers. (C) The system confirms the accepted pose by turning the markers green, followed by automatically triggering the shutter to take the images.

Given the target camera pose (*i*.*e*., optimal pose), our system provides visual guidance displayed in the video feed to allow the users to adjust the camera location. As shown in Fig 6A, four marked corners are showing the intended alignment to the ArUco codes, as well as the yellow arrow indicates the direction and distance the camera should move. We also provide texts and audio guidance for visually impaired people (shown at the bottom of the images in Fig 6). As the user moves the camera closer to the optimal poses, the arrow will become shorter. Once the arrow is short enough, the user can align the red squares on the four corners of the screen to the four ArUco codes on the board to perform the height adjustment until reaching the preset tolerance (as shown in Fig 6B). After this, the red squares will turn to green, and the system will advise the users to hold for one second till it automatically triggers the shutter to take a photo (as shown in Fig 6C). Image content outside of the color board will be automatically cropped to preserve privacy and be rectified to orthogonal views for further image analysis.

### The color correction algorithm

Once the image is captured, we propose a color correction algorithm to perform the color correction using the reference color on the sides of the reference board. We use a linear transformation [39] that transforms the color from the side color bars to their pre-recorded reference values, and the goal is to apply the same transformation to the object of interest in the image (in the sample region). Assuming a linear transformation, we formulate a transformation called simple linear model as in Eq 3.

$$I_{ref}^{c}=\alpha^{c}\times I_{image}^{c}+\beta^{c}+e,\;c\in \{R,G,B\}$$ (3)

where c represents the channel of the image. $I_{ref}^{c}$ refers to the expected color intensity of a point on the color stripe, and $I_{image}^{c}$ refers to the color intensity value from the captured image. α^*c*^ and β^*c*^ are linear coefficients for this linear model, *e* is the error term. However, in an indoor and complex environment, merely using a simple linear model may not yield satisfactory results, as the direction and intensity of light are heterogeneous. Therefore, we propose to model the uneven lighting effects using a spatial varying coefficient model (SVCM) [40] as shown in Eq 4, where the linear coefficients α^*c*^ and β^*c*^ are correlated with the location of the pixels.


$$I_{ref}^{c}(x,y)=\alpha^{c}(x,y)\times I_{image}^{c}(x,y)+\beta^{c}(x,y)+e.\;c\in \{R,G,B\}$$ (4)

**Fig 7:**
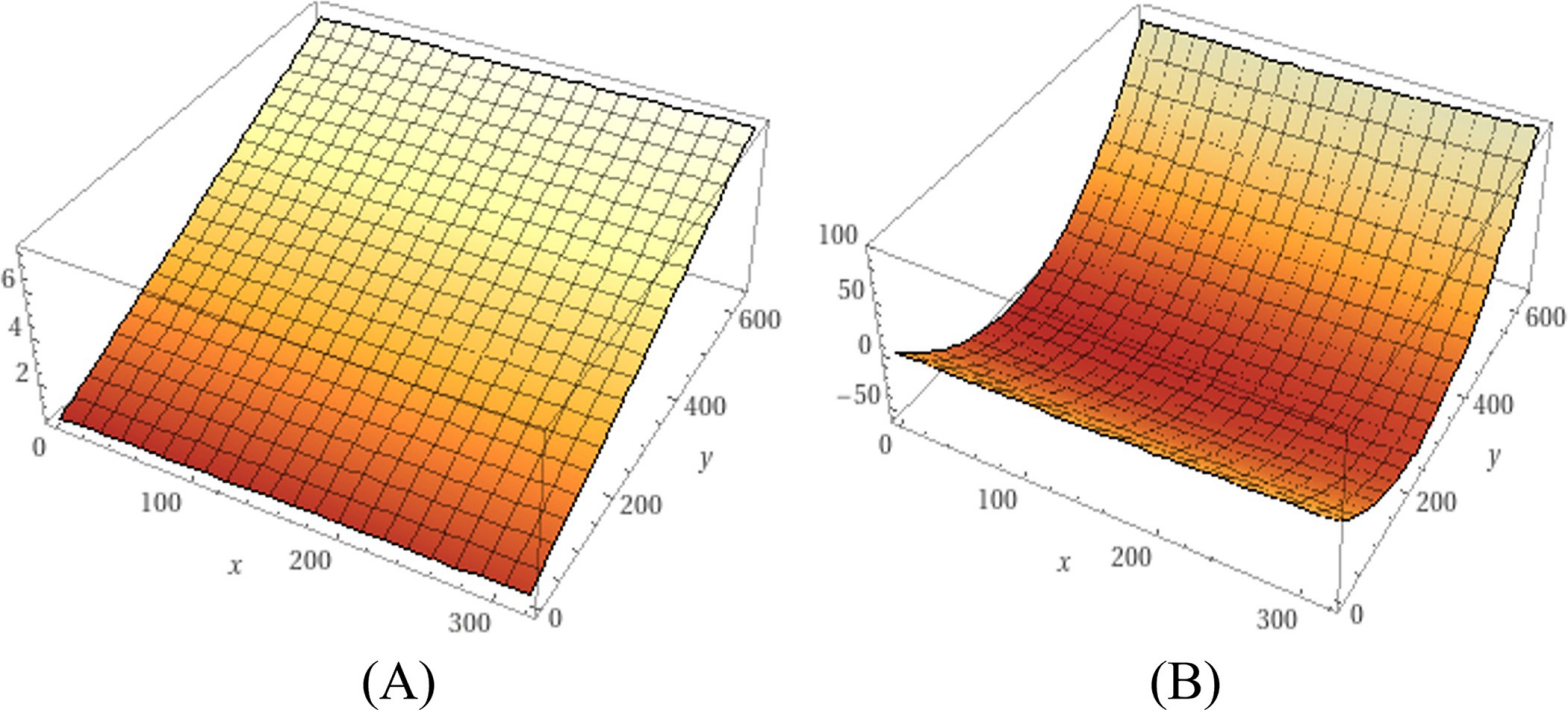
Examples of parametric 2d-spatial varying functions. (A) $f_{1}(x,y)=ax+by+c$, 1^st^ order (primary) surface, 3 parameters. (B) $f_{2}(x,y)=ax^{2}+bxy+cy^{2}+lx+my+n$, 2^nd^ order (quadric) surface, 6 parameters.

To compute the spatially varying coefficient models, we propose to use a first or second order function as shown in Fig 7 to fit α^*c*^(*x*,*y*) and β^*c*^(*x*,*y*), taking the observed color stripes and their reference values as the observations. On the one hand, we assume the light variances can be modeled by a first-order function due to the small physical size of the test badge. On the other hand, this simple and first-order model can produce more robust results and is less likely to produce overfitting problems. More models are tested as reported in Section “Impact of different color stripe patterns and color correction models on the reference board”, including the simple linear model, our spatial varying coefficient models using first and second order functions to fit the coefficients, and non-parametric models.

**Fig 8:**
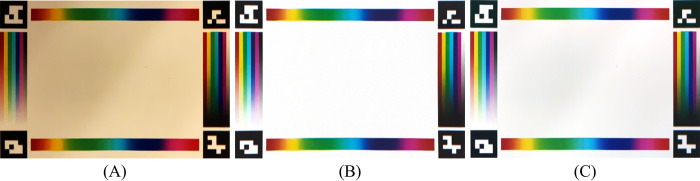
An example of our color correction algorithm (with primary surface function *f*_1_). (A) Cropped and rectified image. (B) Standard color reference board. (C) Corrected image—*f*_1_.

Our spatially varying coefficient models can be easily fitted by using ordinary least squares (OLS) [41]. Specifically, we sampled corresponding pairs of points with 10-pixel intervals on the color stripes on both captured image and standard color reference board (digitalized from printed color reference board), in a total of 424 pairs. The colors of those pairs were used to fit the first-order SVCM using OLS. We fitted the model for each channel (red, green, and blue) separately. Then the color of the entire captured image was corrected by applying those 3 models to all the pixels of the corresponding channel. Fig 8 presents an example of color correction: the image is taken under the uneven room light by the smartphone, then cropped and rectified using the method described in Section “Augmented reality-based image capture guidance module” (Fig 8A). It can be seen that there is a gradual change in illumination in the original image (Fig 8A). After applying our correction model, the resulting image (Fig 8C) is shown to be much similar to its reference (Fig 8B).

## Experiments

In this section, we present two simulated experiments and two real (physical) experiments to verify our proposed system. In the simulated experiments, we synthesize an “object of interest” at the center of the color reference board to obtain quantitatively examine the color correction algorithm. In real experiments, firstly, we independently capture an object and compare the difference after the correction to validate the effectiveness of our solution. Secondly, we design a pH test paper reading experiment to demonstrate the practical value of our proposed system.

### Synthetic experiments

**Fig 9:**
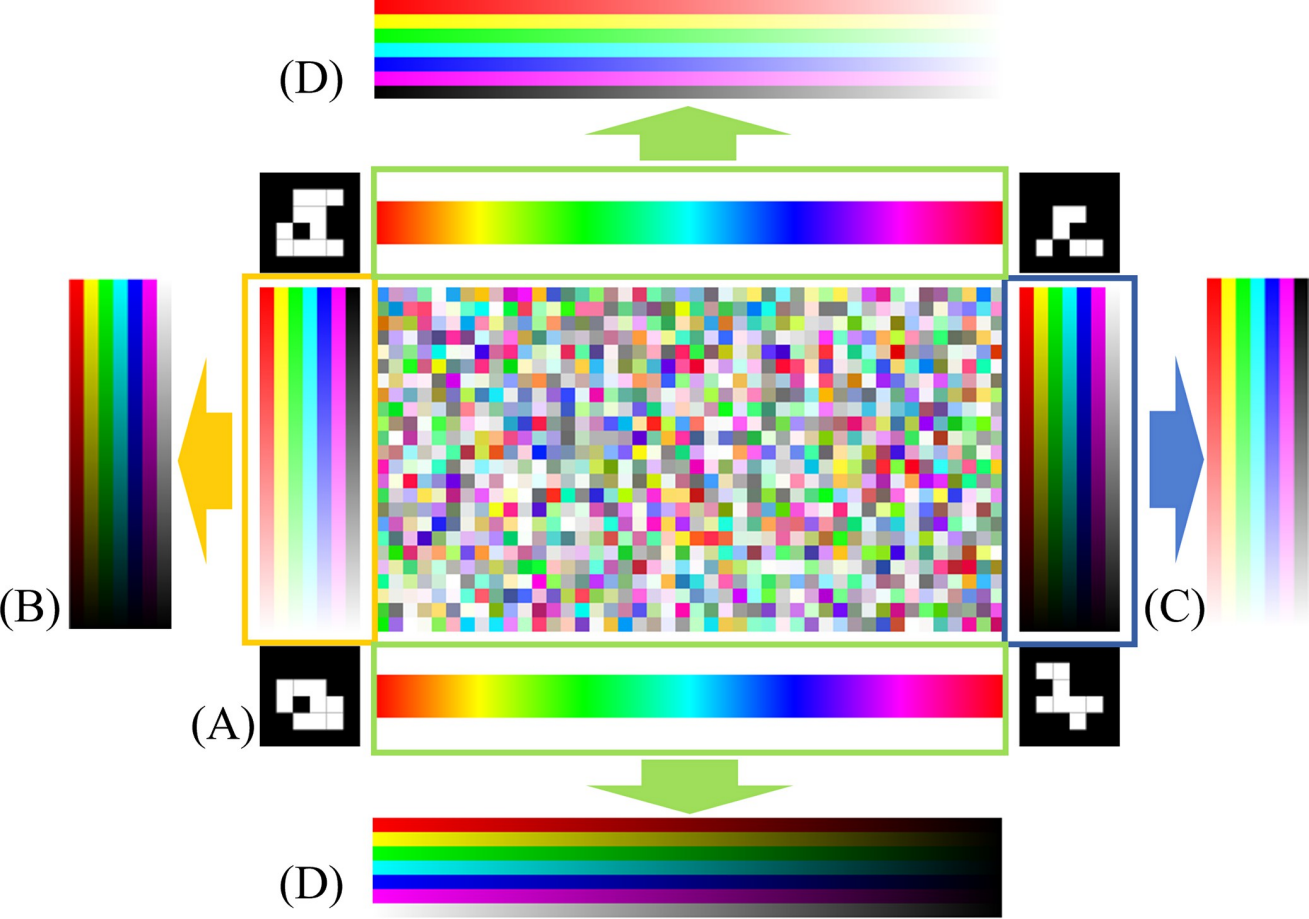
Rendered color mosaic and designs of color reference board. (A) combination of **H**ue, **S**aturation, and **V**alue stripes (B) combination of **H**ue and **V**alue stripes. (C) combination of **H**ue and **S**aturation stripes. (D) combination of **S**aturation and **V**alue stripes.


In this subsection, we evaluated our algorithm with the randomly generated 44 × 24 color mosaics and sampled one pixel for each mosaic to cover the potential color space as shown in Fig 9. We use the mean root mean squared error (mRMSE) over the RGB channels to quantitatively evaluate the performance of our algorithm (Eq 5). Compared to other metrics, such as mean absolute percentage error (MAPE), RMSE measures the absolute differences and does not impose a biased assessment for different color values, which is preferred in our experiments. Then we tested different combinations of color stripe patterns in our badge design and color correction models to understand if other variants of combinations of color patterns and color correction models may lead to better results. Additionally, we ran a simulated experiment by finding the optimal pose of the camera for correction, all using our synthesized “object of interest”.

$$\begin{array}{c} mRMSE=\frac{1}{3}\sum_{c}^{c\in \{R,G,B\}}RMSE^{c},\\ where\;RMSE^{c}=\frac{1}{\sqrt{W\times H}}\sqrt{\sum_{x}^{W}\sum_{y}^{H}{\left(I_{ref}^{c}(x,y)-I_{corr}^{c}(x,y)\right)}^{2}},\;c\in \{R,G,B\} \end{array}$$ (5)

where W and H are the width and height of the image, $I_{ref}^{c}$ is the pixel value of channel *c* on the image pixel value of the standard color reference board, respectively, $I_{corr}^{c}$ is the value of the color-corrected image.

#### Impact of different color stripe patterns and color correction models on the reference board

**Fig 10:**
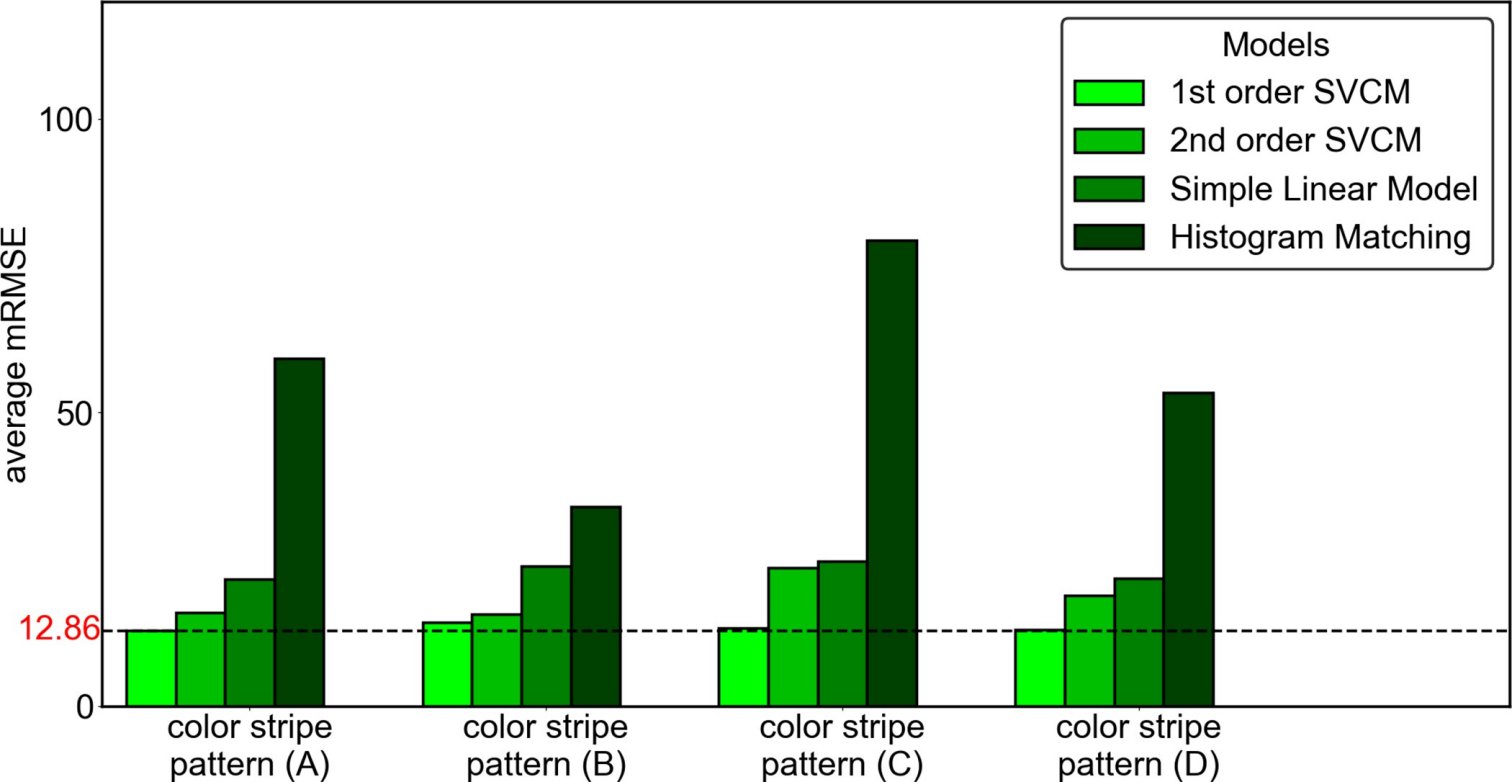
The performance of different combinations of color stripe patterns (A-D) and models.

In this experiment, we compared three variants of patterns of the color stripes and four-color correction models to study 1) the sensitivity to results for different color stripe patterns, and 2) other correction models in addition to the linear SVCM model. The performance is evaluated by mRMSE computed from the generated color mosaic (Fig 9). The color reference board (design (A) in Fig 9) presented in previous sections consists of three kinds of color stripes groups: the **S**aturation stripes on the left side in the yellow rectangle (*H*∈[0,1], *S*∈[0,1], *V* = 1); the **V**alue stripes on the right in the blue rectangle (*H*∈[0,1], *S* = 1, *V*∈[0,1]) and the **H**ue changing stripes on the top and bottom in the green rectangle (*H*∈[0,1], *S* = 1, *V* = 1). We derived three variants of the patterns with different combinations as shown in Fig 9: (B) The variant with Saturation fixed stripes (C) The variant with **V**alue fixed stripes and (D) The variant with the Hue fixed stripes. As for color correction models, we compare the 1st order, 2nd order spatial varying coefficient model (SVCM) with a simple linear model without spatial varying coefficient and a non-parametric method called histogram matching [42]. Results are presented in Fig 10. The image is taken under 3 different color temperatures (2800k, 4000k, and 6500k) and with 3 different lighting directions, and we take the average mRMSE from 9 readings.

From Fig 10, we can see that the color stripe pattern (A) with 1^st^ order spatial varying coefficient model achieves the least mRMSE = 12.86. which we use for the rest of the experiments. Pattern (A) has all the components of the HSV space that encapsulate the full range of colors. This is evidenced by the fact that generally, all the models perform the best for the pattern (A), with the exception that the histogram matching method performs variably with different designs but is poorer than the other models. The parametric models perform similarly with pattern (A) and pattern (D), with pattern (D) marginally better, meaning that the **H**ue channel is least informative for color correction.

#### Optimal viewing angle

**Fig 11:**
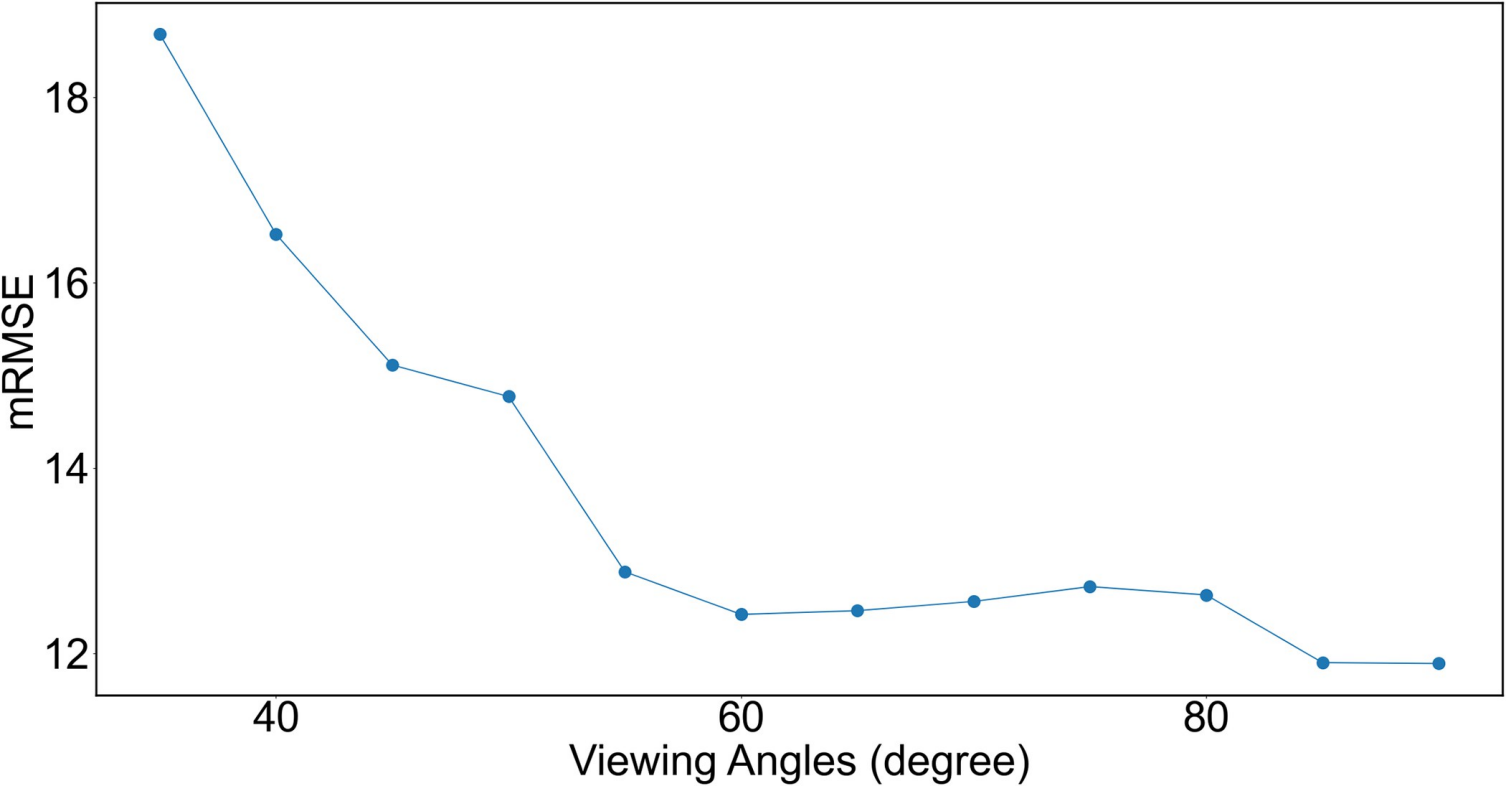
The curve of mRMSE vs viewing angles. mRMSE over RGB channels gradually decreases as the viewing angle increases. 90 degrees means the camera is right above the board.

In this section, we analyze the effect of different viewing angles on color correction accuracy to determine the optimal viewing angle in our AR guiding submodule. We render images with viewing angles from 35 to 90 degrees with a 5-degree step, for each image, we evaluate its mRMSE using its ground truth color to understand how the accuracy changes with respect to the angles. During the experiments, we assume a Lambertian surface (the most common one in the natural world), and the results are shown in Fig 11. As can be seen, the accuracy increases almost monotonically as the view angle approaches 90 degrees.

#### Comparison with other colorimetric measurement methods

In this section, we compared the traditional color correction method using a color checker called “color checker correction” and two recent colorimetric measurement methods mentioned in the Section “Introduction” with our proposed system.

Traditional correction method associated with a color checker uses the simple linear model in Eq 3 to achieve color correction. The difference is that the $I_{ref}^{c}$ refers to the expected color value of a patch on the color checker, and $I_{image}^{c}$ refers to the color value from the captured image of the color checker. This kind of correction does not account for heterogeneous lighting effects over different parts of the object. A color checker may work to a degree that the corrections are accurate in the vicinity of the color checker. As an analogy, our color correction algorithm with the color reference board is close to using multiple color checkers in the space and applying different correction coefficients for each. Essentially this is done by using full color-space with a spatial varying function to ensure continuity in the functional space. In this experiment, we sampled only 24 points from the stripes to simulate the color checker.

Both recent colorimetric measurement methods involve the use of specific hardware and software, so reproducing them exactly can be challenging. We attempted to conduct the comparative study as fairly as possible. Since codes are not available for these methods, we reimplemented them based on the paper. One of them is from [2], where the authors used the flashlight on the smartphone as the dominant light source and rescaled the color with the white and black background, notated as “Flashlight rescale”. The other is from [21], where the flashlight was used to remove the environmental lighting, and the color checker was used to fit a mapping from RGB to CIE XYZ space to achieve device-independent, notated as “Flashlight color checker correction”. As for the Flashlight color checker correction method, the system was simplified by removing the Intensity Non-Uniformity Correction (INUC). In addition, after transferring the RGB values to the CIEXYZ space with fitted mapping, the values were converted back to RGB for evaluation and visualization with the default transformation between RGB and CIEXYZ.

In this experiment, we took the images of our printed color reference board under a fluorescent lamp in the lab with the iPhone SE2 and did not consider the different devices. For both recent methods which used a flashlight as an additional light source, we took the images perpendicular to the color reference board (right above the board). Then the region with strong reflection, which looked like a bright white spot at the center, was ignored during evaluation. For all methods, the standard colors for evaluation were from the digitalized printed color reference board. The mRMSE and images for different methods are shown in Table 1.

**Table 1:** Comparison of different colorimetric measurement methods.

Method	mRMSE
Color checker correction	13.93
Flashlight rescale [2]	14.48
Flashlight color checker correction [21]	67.86
Ours	13.41

Compared to the Color checker correction method which was built on our color reference board with a simulated color checker from the stripes. our method with the first-order SVCM can address uneven lights on the objects and achieve lower mRMSE. The Flashlight rescale method has higher mRMSE than ours. In addition, this method relies much on the manual selection of the black and white reference points. The Flashlight color checker correction method has much higher mRMSE on the validation points. The possible reason for this is that the assumption that the response of sensors across the three channels is linear with increasing intensity is not always achievable or too strict to fulfill. Compared to those recent methods, our system is much more user-friendly with much more flexibility and better correction performance.

### Real-world experiments

We evaluate the performance of our color correction algorithm through two real-world experiments: 1) device-independent object color correction under varying lighting conditions, and 2) a pH reading experiment comparing our colorimetric pH measurement algorithm with human eye readings.

#### Device-independent color correction

**Fig 12:**
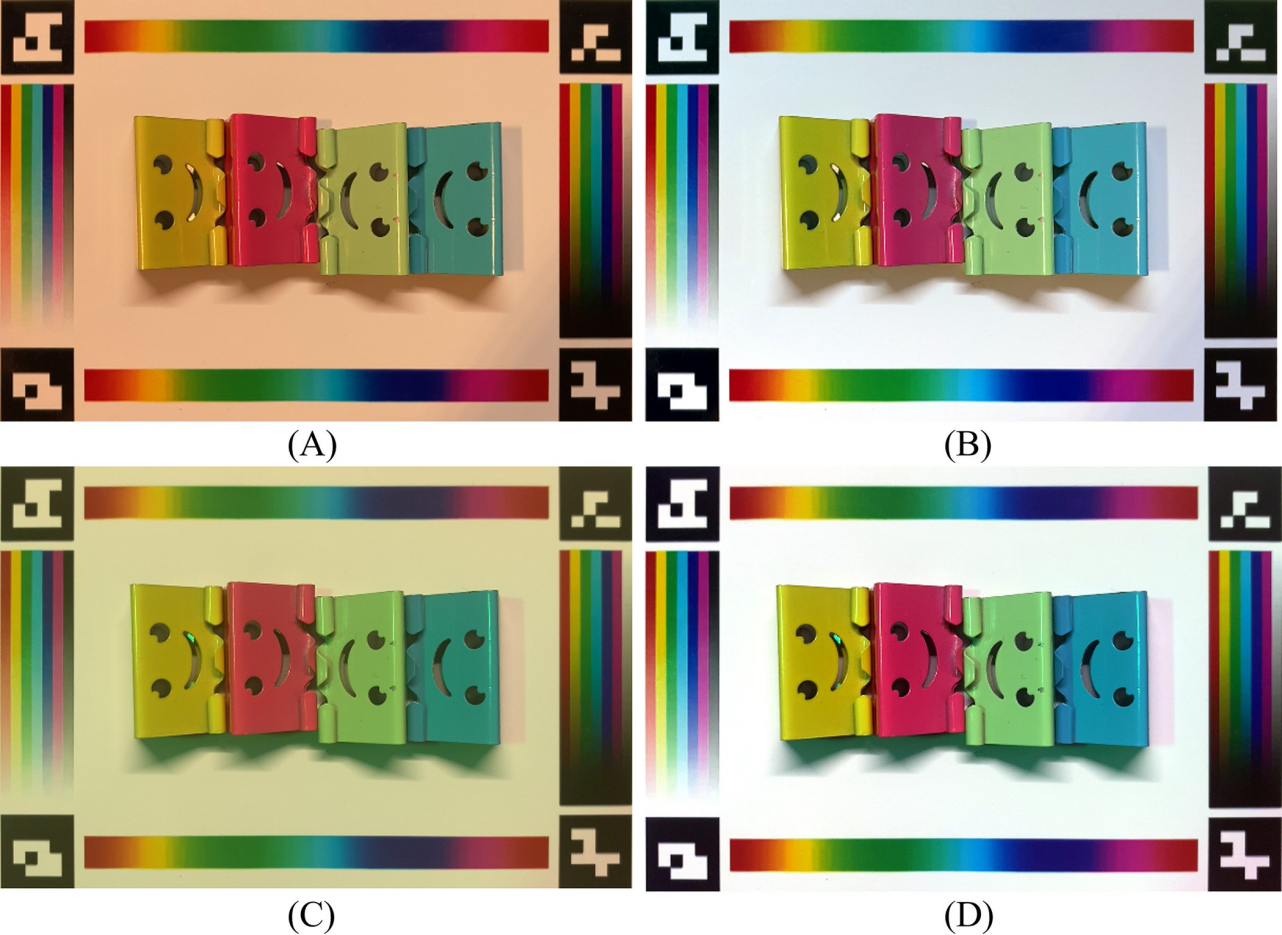
Example images under different lights and camera and their correction results. (A) and (C) images were taken under two different mixed lights by iPhone SE 2^nd^ generation and iPhone XSMAX, respectively. (B) and (D) the corresponding images after color correction.

**Fig 13:**
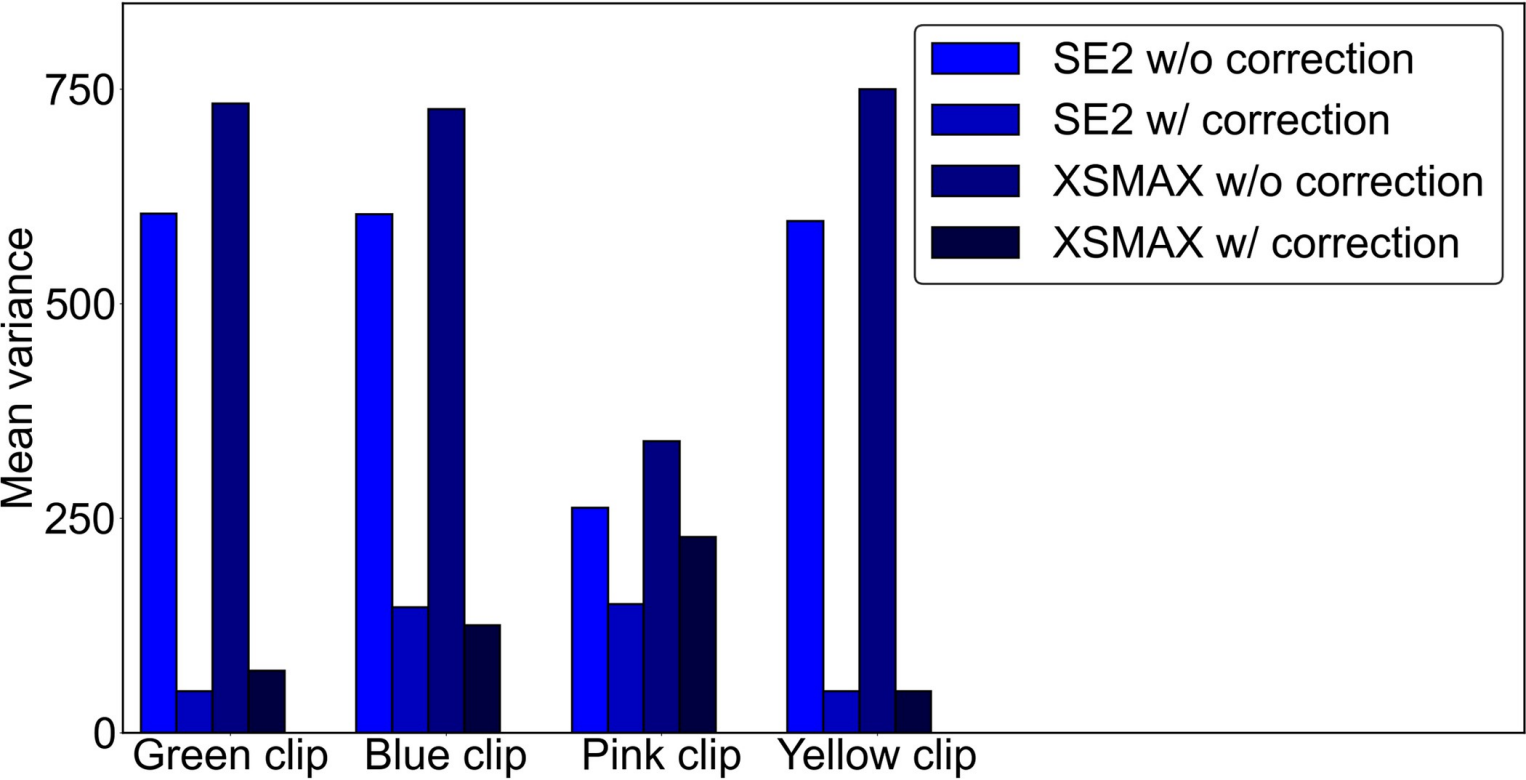
Color correction performance of different binder clips.

In this experiment, we took images of objects with cameras of two mobile phone models (iPhone SE 2^nd^ generation (released in 2020) and iPhone XSMAX (released in 2018)) under 15 different lighting conditions. The goal is to measure the color differences of the object under different lights and cameras before and after the color correction. Ideally, we expect the corrected colors of the objects to be consistent despite the original images being captured under different lighting conditions and cameras. To facilitate the evaluation, we used a few binder clips with distinctively different colors lined up in a row, such that the before- and after-correction can be easily quantified. Two examples of these uncorrected images are shown in Fig 12A and 12C, and their respectively corrected images are shown in Fig 12B and 12D. The strong visual comparison demonstrated that the correction algorithms can yield visually consistent images of the same objects, despite these images being taken under distinctively different lighting conditions and cameras. We computed the variance of the 15 images from each smartphone, for each color clip. The same variance is calculated for images after the color correction. It was shown in Fig 13 that the corrected images have a much smaller variance, approximately at a factor of up to 15 times. We also observed that the level of improved color consistency is correlated with the color to be corrected, for example, the pink clip has less improvement than the other three, which might be due to its already small color variance before the correction.

#### Comparison of our colorimetric pH measurement algorithm with human eye readings of pH stripes

*Experiment setup*. We designed a pH test paper reading experiment to quantitatively compare our colorimetric pH measurement algorithm with human eye readings. In this experiment, we tested six pH buffers (3.0, 6.86, 7.0, 7.8, 9.0, and 9.18), and 3 different kinds of standard pH test paper and reference color chart covering pH ranges of 3–5.5, 6–8, and 8–9.5 (Fisher Scientific, Pittsburgh, PA), separately. Except for the buffer with pH 7.8, all other buffers were commercially obtained colorless reference buffers (Fisher Scientific, Pittsburgh, PA). The buffer with a pH of 7.8 was prepared by combining 3.68 mL 1 M potassium phosphate dibasic (K_2_HPO_4_, CAS# 7758-11-4), 1.32 mL 1 M potassium phosphate monobasic (KH_2_PO_4_, CAS# 7778-77-0) and 45 mL DI water (18.2 MΩ**ּ** cm) [43]. The pH of the buffer was measured with an Orion 5-Star portable meter equipped with a pH combination electrode (Cat. no. 9107APMD, Fisher Scientific, Pittsburgh, PA), and adjusted as necessary with 1M potassium phosphate dibasic or monobasic. We invited six participants without self-reported visual impairments related to color perception to do the human eye readings; their ages were estimated to be between 18–40 years old. The human eye reading vs colorimetric pH measurement algorithm experiment was organized as follows.

The entire experiment was carried out on a large laboratory bench under bright fluorescent light. Freshly poured aliquots of the pH buffers were placed behind a screen to obstruct them from the view of the participants during preparation. The participants were allowed to enter, received instructions, and lined up to take readings. For each of the six trials lasting approximately 3–5 minutes, the pH paper was dipped into the unknown buffer while obstructed from view, placed in the center of a color reference board, photographed with the iPhone SE 2^nd^ generation, and then shown to the participants for pH estimation. Participants read the pH values of pH paper by comparing the color of pH test papers to the reference color chart. The readings were performed individually by each participant without sharing or discussing results with the others. To minimize bias, the sequence of the pH buffers was arranged such that buffers with values close to one another were not read consecutively (*e*.*g*., pH 9 and 9.18), and the participants were instructed to line up in random order for each reading. In parallel, based on our color correction system, we designed a colorimetric pH measurement algorithm that will read the color from the images of the pH test paper and the reference color chart. Then the algorithm will measure the pH by comparing colors (details in the next subsection). To minimize light and shadow inter-trial variability, each photograph and human reading were collected in the same respective locations on the bench *i*.*e*., the light condition was kept the same. So, we also call this experiment the reference case.

We also did an additional experiment without human readings where the images of the reference color chart were taken under a different light condition (outside sunlight) from where the images of pH test paper were taken (in the laboratory). In this additional experiment, we showed that our color correction system can improve the colorimetric measurement accuracy when the color changes under different light conditions. This characteristic has much practical value in that manufacturers do not need to offer the physical color reference chart but can encode a digital copy of the color reference in the mobile app. It not only saves the user’s reading step but can standardize the reading process to improve accuracy. During actual usage, users just need to take an image of the pH test paper using the mobile phone, the color will be corrected, and an accurate pH value will be measured. Thus, we call this additional experiment color chart free case. This characteristic can also facilitate many other colorimetric measurement applications.

The above experiments were approved by the Ohio State University Institutional Review Board, study number 2022E0482. Consent was obtained via an online unsigned script to avoid linking participants to their responses. Participants checked “yes” to consent and then entered their readings in an online survey on the next page.

**Fig 14:**
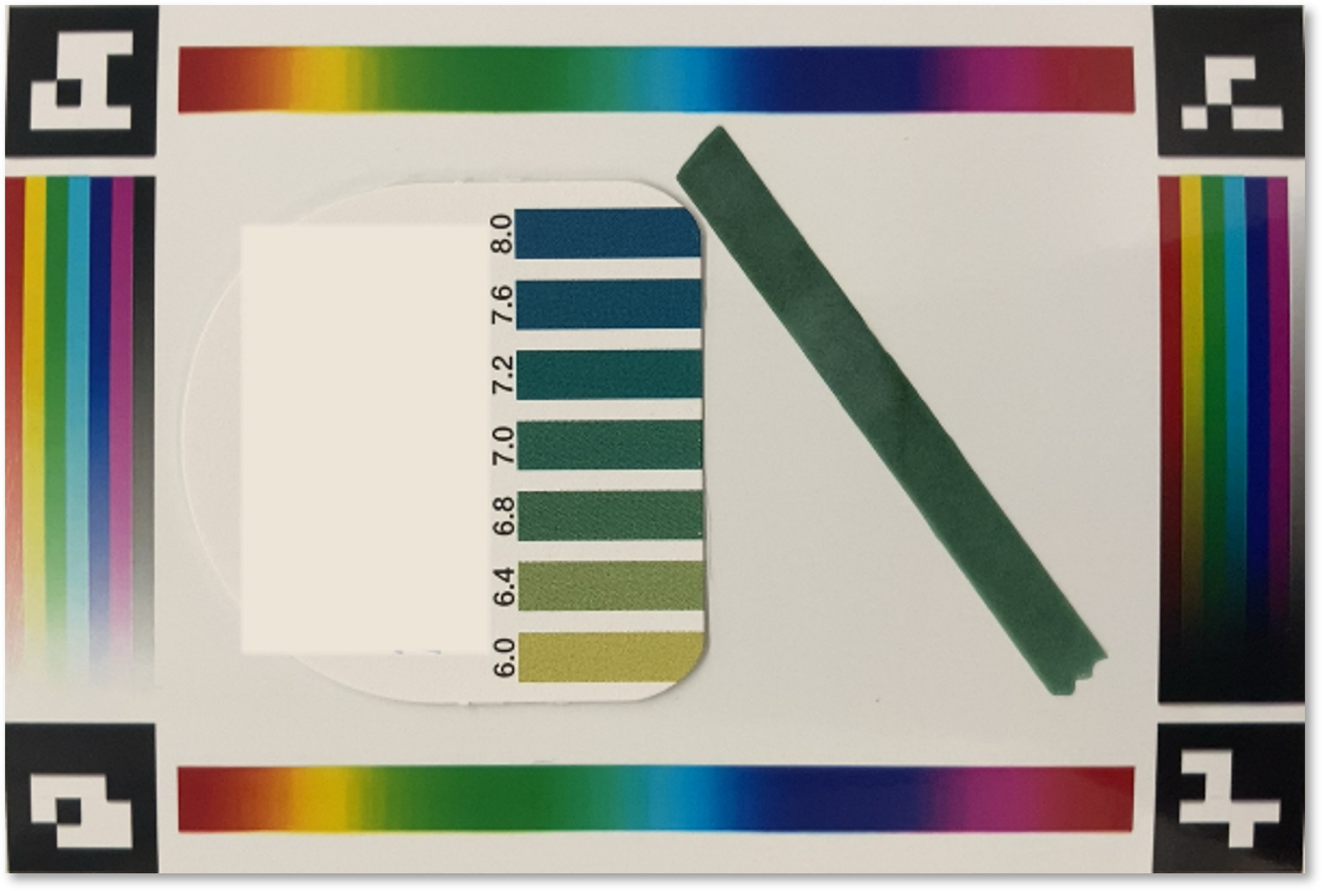
An example of cropped and rectified image of a reference card and test paper. Left: reference card. Right: the test paper reacted with a sample whose pH = 7.0. Here we blocked the brand of the test paper with a white box over that part of the image.

**Fig 15:**
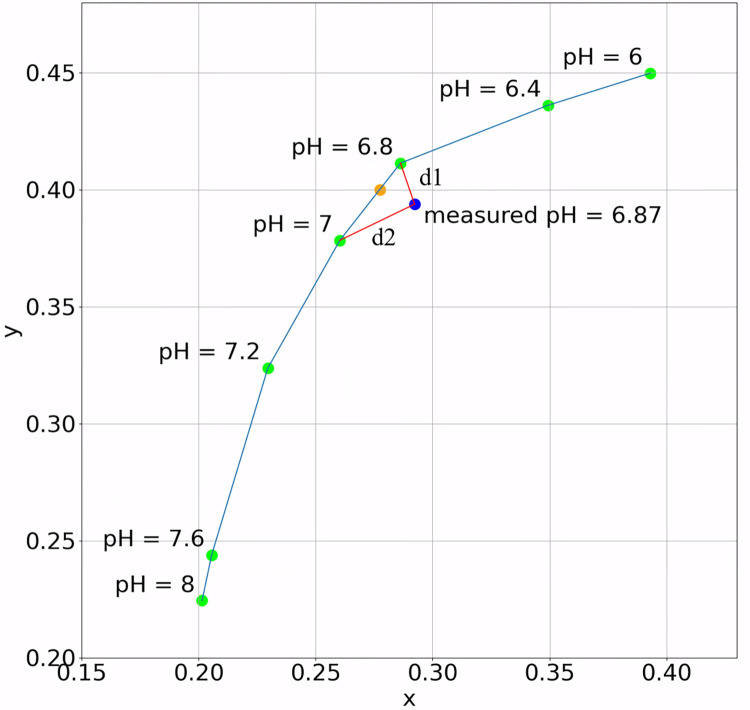
pH value interpolation method. The curve was built from the reference color chart (blue line). The measured color of reacted pH test paper may not perfectly lay on the reference curve (point outside curve). We find the distances to the two closest reference points (pH = 6.8 and 7), d1 and d2. Then interpolate the pH value by applying the IDW method: measured pH = (6.8 × d2+7 × d1)/(d1+d2). Then split the line segment from 6.8 to 7 with the ratio d1/d2 to get the point of the final measured pH value on the curve.

*Colorimetric pH measurement algorithm*. For example, Fig 14, is an image of pH test paper reacted with the solution which a pH value is 7.0, and the corresponding color reference chart covers the range from 6–8. Since the color chart only resolves discrete pH values (at an interval/resolution of 0.2–0.4), to determine the color beyond this resolution, we interpolate the pH value of the measured color of the pH test paper by using the inverse distance weighting (IDW) method [44] to the two closest data points on the color chart. This process is shown in Fig 15: each green point presents the reference color to a pH value on the color chart. The blue point is a measured color from the pH test paper, by determining its color difference to each of the reference colors on the color chart, we find the nearest two reference points (green), linked to the measured point (blue) via red lines. A weighted average is computed to determine the final measured pH value (orange point) that lies between the two reference points, and the weights are inversely proportional to the color difference. Following the most common practice in colorimetric pH test paper reading [21, 45], we use chromaticity x and y derived from International Commission on Illumination (CIE) 1931 XYZ color space [46] to define the color reference curve and interpolate for measurements. Using this process, we are able to determine pH values beyond the color chart resolution.

*pH reading experiment results*. The experiment results are reported in Table 2. It includes the results of two experiments, 1) reference case, where the color chart and the pH test paper are captured together in the laboratory where participants read the pH. In this case, our colorimetric pH measurement algorithm is compared with human eye readings; and 2) the color chart free case, where the color charts and the pH test paper are separately captured. In this case, we compared the performance of our algorithm when the input images are from similar or dramatically different light conditions (laboratory vs outside sunlight).

**Table 2:** pH errors and values measured by human observers and smartphone apps with and without our correction algorithm under different lighting conditions.

	Reference–pH test paper and color chart co-located (color chart and pH test paper under the same illumination)			Color chart free pH reading (color chart and pH test paper under different illumination)	
Ref. pH value	w/o correction	w/ correction	avg. human readings	w/o correction	w/ correction
**3.00**	+0.16 (3.16)	+0.15 (3.15)	**-0.07** (2.93)	+0.16 (3.16)	**+0.13** (3.13)
**6.86**	-0.17 (6.69)	-0.20 (6.66)	**-0.06** (6.80)	-0.24 (6.62)	**-0.20** (6.66)
**7.00**	-0.10 (6.90)	-0.13 (6.87)	**-0.08** (6.92)	-0.10 (6.90)	**-0.09** (6.91)
**7.80**	**+0.05** (7.85)	+0.07 (7.87)	+0.17 (7.97)	+0.09 (7.89)	**+0.06** (7.86)
**9.00**	**+0.13** (9.13)	-0.15 (8.85)	+0.45 (9.45)	-0.22 (8.78)	**-0.20** (8.80)
**9.18**	+0.12 (9.30)	**0.00** (9.18)	+0.67 (9.85)	-0.06 (9.12)	**-0.04** (9.14)
**MAE**	**0.12**	**0.12**	0.37	0.15	**0.12**

From the results of the reference case, in general, human readings performed well on solutions that have a clear reference reading in the color charts (*e*.*g*., solutions of 3.00, 6.86, 7.00), but worse on reading pH test paper of solutions beyond the resolution of the charts (*e*.*g*., solutions of 7.80, 9.18). As a result, the human readings achieved a Mean Average Error (MAE) of 0.37. In contrast, the readings determined by our algorithm show stable performance on all solutions and achieved a Mean Average Error of 0.12, three times better than human reading. From the results of chart free case, we observed that the Mean Average Error of the pH reading decreased from 0.15 to 0.12 as our color correction algorithm is applied. This error is also consistent with the reference case where the color charts and the pH test papers are taken under the same illumination.

Those observations conclude that firstly, our proposed system can achieve approximately tripled accuracy as compared to human eye readings. Secondly, it also has the ability to extrapolate reading beyond the resolution of reference charts. Thirdly, it can accurately measure color under different lighting environments and the manufacturer does not need to offer a physical color reference chart to users.

Due to the high prevalence of visual impairment, which affects some 285 million people worldwide [47], our sample of research participants represents an underrepresentation of human accuracy issues and our solution improves the accessibility of colorimetric measurement for people with visual disabilities.

## Conclusions

In this paper, a novel smartphone-based solution for accurate colorimetric measurement is proposed. It consists of a novel color reference board, with an augmented-reality (AR) guiding system and a novel color correction algorithm. The color reference board is designed to cover the full visible color space to provide an absolute reference of colors to determine the color values. The AR guiding system introduces the “human-in-the-loop” process to capture images with the desired camera position and viewing angle to reduce the impact of various lighting reflecting effects. A novel color correction algorithm with the first-order spatial varying coefficient model is proposed to couple the color stripes on the reference board, to provide effective color corrections to recover the colors distorted by the device and environmental lighting. Both simulated and real data experiments are performed, which include testing samples simulated through computer graphics-based rendering, real object color correction as well as pH reading from color stripe kits. These experiments suggest that our proposed system is able to capture color consistent images under varying lighting environments and devices, which effectively reduces the color variances up to a factor of 15. Specifically, we showed that in our pH reading experiment, regardless of varying lighting conditions, our system can achieve pH readings three times more accurately than human reading and can effectively determine pH values that are beyond the resolution of the reference color chart of the pH test kits.

Overall, these improvements in color determination have broad implications for improvements in a wide range of applications, including medical diagnostic tests, environmental monitoring, and agricultural applications. Our system also improves the accessibility to accurately read colors for those with visual impairment. Our future work will consider developing more advanced color correction models that address partial shadow problems at data capture in cluttered environments, as well as extend the current system to an Android implementation for scalability and enabling more applications. We will also apply and adapt our system to broader applications such as medical diagnostician tests, environmental monitoring, and agricultural applications.
